# Characterizing the Ultraviolet (UV) Screening Ability of L-5-Sulfanylhistidine Derivatives on Human Dermal Fibroblasts

**DOI:** 10.3390/md23020057

**Published:** 2025-01-24

**Authors:** Alessia Luccarini, Fabio Marcheggiani, Roberta Galeazzi, Annalisa Zuccarotto, Immacolata Castellano, Elisabetta Damiani

**Affiliations:** 1 Department of Life and Environmental Sciences, Polytechnic University of Marche, 60131 Ancona, Italy; a.luccarini@pm.univpm.it (A.L.); r.galeazzi@univpm.it (R.G.); 2 Department for the Promotion of Human Sciences and Quality of Life, San Raffaele Roma University, 00166 Rome, Italy; fabio.marcheggiani@uniroma5.it; 3 Department of Molecular Medicine and Medical Biotechnology, University of Naples “Federico II”, 80131 Naples, Italy; annalisa.zuccarotto@unina.it; 4 Department of Biology and Evolution of Marine Organisms, Stazione Zoologica Anton Dohrn, 80121 Naples, Italy

**Keywords:** photoprotection, ovothiol analogs, sulfur-containing amino acids, ultraviolet radiation (UVR) damage, natural UV filters, human dermal fibroblasts

## Abstract

Using sunscreens is one of the most widespread measures to protect human skin from sun ultraviolet radiation (UVR) damage. However, several studies have highlighted the toxicity of certain inorganic and organic UV filters used in sunscreens for the marine environment and human health. An alternative strategy may involve the use of natural products of marine origin to counteract UVR-mediated damage. Ovothiols are sulfur-containing amino acids produced by marine invertebrates, microalgae, and bacteria, endowed with unique antioxidant and UV-absorption properties. This study aimed to evaluate the protective effect of synthetic L-5-sulfanyl histidine derivatives, inspired by natural ovothiols, on human dermal fibroblasts (HDFs) upon UVA exposure. By using a custom-made experimental set-up to assess the UV screening ability, we measured the levels of cytosolic and mitochondrial reactive oxygen species (ROS), as well as cell viability and apoptosis in HDFs, in the presence of tested compounds, after UVA exposure, using flow cytometry assays with specific fluorescent probes. The results show that L-5-sulfanyl histidine derivatives display a UV screening capacity and prevent loss in cell viability, the production of cytosolic and mitochondrial ROS induced by UVA exposure in HDFs, and subsequent apoptosis. Overall, this study sheds light on the potential applications of marine-inspired sulfur-containing amino acids in developing alternative eco-safe sunscreens for UVR skin protection.

## 1. Introduction

Starting during the Industrial Revolution, the continuous emission of carbon dioxide and gas pollutants in the atmosphere has contributed to the thinning of the ozone layer and the penetration of the sun’s ultraviolet radiation (UVR) into the stratosphere. UVC rays (<280 nm), and most of the UVB ones (280–320 nm), are shielded by the ozone layer, whereas ~5% of UVB and ~90% of UVA (320–400 nm) rays reach the Earth and induce oxidative stress that leads to cell damage in living organisms [1]. In humans, exposure to sunlight is essential for the conversion of 7-dehydrocholesterol in pre-vitamin D3 in the skin [2]. However, excessive exposure to UVR causes sunburns, photoaging, and increases the risk of developing skin cancer [3,4]. Consequently, the World Health Organization has recommended avoiding sun overexposure during peak hours and using sunscreens to protect skin from the harmful effects of UVR [5,6]. Commercial sunscreens contain inorganic or organic compounds endowed with UV-filtering properties (e.g., zinc oxide, titanium dioxide, benzophenone-3, and octylmethoxycinnamate) to prevent sunburn, skin aging, wrinkle formation, and skin cancer [6,7]. Nevertheless, current UV filters have several limitations. These include stability issues (as, upon UV exposure, the filters can degrade and their effectiveness and ability to screen out harmful UV rays can reduce over time, as in the case of tert-butylmethoxydibenzoylmethane [8]); skin sensitivity and allergic reactions (as in the case of benzophenone-3, which is a contact and photocontact allergen [9]); broad-spectrum protection (since most UV filters do not provide a balanced protection across the entire UVB and UVA spectrum); environmental and health impacts (as in the case of benzophenone-3 and octylmethoxycinnamate, as the sunscreens that contain them have been banned from sale and distribution in Hawaii, Key West in Florida, and other islands and nations for their suspected harm to coral reefs and marine organisms, in which they can induce endocrine disruption and hepatotoxicity [10,11,12,13,14]). Also, today’s consumers are more aware of the origin and eco-sustainability of their personal care products and are willing to pay more for products and brands that align with their personal values. Therefore, natural products could overcome some of the limitations outlined above and are gaining traction as alternatives to synthetic chemicals in the cosmeceutical sector [5,15].

Photoprotection is the biochemical process that helps organisms cope with molecular damage caused by sunlight, and recent studies indicate that several natural compounds exert photoprotective effects on the skin. This is achieved not only through their direct UV-absorption properties but also through their antioxidant action by scavenging reactive oxygen species (ROS), as well as by regulation of UV light-induced gene expression, modulation of stress-dependent signaling, and/or suppression of cellular responses like inflammation [15]. The mechanism by which natural compounds act as sunscreen molecules has not been completely elucidated. However, a conjugated π system has been reported to play a key role in protecting genetic material and macromolecules like proteins and lipids within organisms [16]. For example, mycosporine-like amino acids (MAAs) synthesized by cyanobacteria and microalgae to protect themselves against UVR damage, are UV-absorbing/UV-screening compounds due to their chemical structure and extinction coefficient value, and are photostable, making them long-term protectants [17,18]. Furthermore, these compounds also provide broad-spectrum UV protection, absorbing a wide range of UV wavelengths and offering better overall skin protection compared to some synthetic UV filters [19]. Unlike many synthetic UV filters, natural marine-based UV filters tend to be biodegradable and non-toxic to marine life, making them a sustainable choice for partially replacing harmful chemicals such as benzophenone-3 and octylmethoxycinnamate [20]. Several peptides isolated from other marine organisms have also demonstrated protective activity against UV damage. For example, the antioxidant peptide extracted from the seahorse *Hippocampus abdominalis* SHP2 was shown to enhance the cell viability of human UVB-stimulated keratinocytes (HaCaT) and dermal fibroblasts (HDFs) by reducing apoptosis and intracellular ROS levels, improving collagen synthesis, and inhibiting matrix metalloproteinases (MMPs) secretion [21]. Similarly, antioxidant peptides isolated from Skipjack tuna (*Katsuwonus pelamis*) cardiac arterial bulbs, TCP3 (PKK), TCP6 (YEGGD), and TCP9 (GPGLM) were reported to restore mitochondrial membrane potential and activate the nuclear erythroid 2-related factor 2 Nrf2/HO-1 pathway, enhancing antioxidant enzyme activity and reducing ROS and lipid peroxidation upon UVB-induced damage in HaCaT cells [22]. Analogous protective properties on UVB-irradiated HaCaT cells were observed for antioxidant peptides purified from *Pinctada martensii* meat hydrolysates which were shown to downregulate the expression of mitogen-activated kinases like p38, extracellular signal-regulated kinases ERK, c-Jun N-terminal kinases JNK, interstitial collagenase and a matrix lysing enzyme [23], thus contributing to inhibiting photoaging. Also, gelatins derived from the cartilage of Siberian sturgeon (*Acipenser baerii*) and Skipjack tuna (*Katsuwonus pelamis*) demonstrated protective properties against UVA-induced damage in human skin fibroblasts by boosting the activity of antioxidant enzymes [24,25]. Lastly, besides their UV-absorbing properties, marine-derived compounds, particularly from algae, can have moisturizing and soothing effects, which might reduce irritation or allergic reactions often associated with synthetic sunscreen ingredients [26].

From the above, it is clear that nature-based compounds represent an attractive and safer option for partially replacing synthetic UV filters in developing eco-safe sunscreens [17,18]. Although synthetic UV filters cannot be completely replaced by natural ones in the immediate future, the information collected so far in the literature provides the basis for furthering studies that aim to develop more efficient and safer sunscreens. In this context, we recently focused our attention on sulfur-containing histidine derivatives, named ovothiols, produced by marine invertebrates, like sea urchins and mollusks, and microalgae, especially diatoms [27,28]. These compounds occur in nature under three differently methylated forms (A, B, and C); they are characterized by unique redox properties [27] due to the position of the sulfhydryl group on the imidazole ring of histidine [29,30,31]. The pKa of the thiol group of ovothiol A is much lower (~1.4) compared to that of glutathione (GSH) (~8.7), and the disulfide of ovothiol A is less stable than the disulfide of GSH [27]. Therefore, ovothiol A and GSH are believed to cooperate to protect cells against ROS-induced damage [27]. Recent studies have reported that the administration of ovothiol A and its precursor 5-thiohistidine in human-inflamed keratinocytes affect ERK and JNK signaling, promoting the translocation of Nrf2 into the nucleus, hence inducing an anti-inflammatory response [32]. The anti-inflammatory action of these compounds has also been observed in human skin tissues taken from individuals undergoing cosmetic surgery [32]. Overall, these findings suggest a protective action for cells and tissues, such as those found in skin.

In the present study, we thus aimed to characterize the UV-screening properties of L-5-sulfanylhistidine disulfide derivatives whose chemical structure is inspired by marine ovothiols for their potential use in nature-based sunscreens and personal care products that may be more eco- and human-safe. We focused on ovothiol analogs due to the following chemical and biological features: (a) antioxidant activity [29,30,31]; (b) characteristic UV-absorption profiles within the desired UV range [33]; (c) biosynthetic regulation by full spectrum visible light in microalgae [34] and transcriptional regulation of the biosynthetic gene by UVR in sea anemones [35]; (d) the presence in the lens of certain fish, suggestive of photoprotective activities [36,37].

We first characterized the absorbance profile of chemically synthesized L-5-sulfanylhistidine disulfide derivatives: 2-amino-3-(5-sulfanylimidazol-4-yl)propanoic acid, hereafter referred to as L-5-sulfanyl histidine or 5-thiohistidine (5-thio); 2-amino-3-(1-methyl-5-sulfanylimidazol-4-yl)propanoic acid, hereafter referred to as iso-ovothiol A (iso-ovoA); and 2-(methylamino)-3-(5-sulfanylimidazol-4-yl)propanoic acid, hereafter referred to as L-*N*-methyl-5-thiohistidine (me-5-thio) [38], when exposed to UVA light and compared them with the natural ovothiol A purified from sea urchin eggs [39]. We also provided evidence of the shielding activities of these compounds on HDFs, showing that they protect cells against the overproduction of cytosolic and mitochondrial ROS induced by UVA rays that can reach the dermal skin layer. Finally, we observe their efficacy in preventing the impairment of cell viability and apoptosis, which are the first events implicated in cellular aging.

## 2. Results

### 2.1. Absorption Spectra of Marine Ovothiol and Its Chemical Analogs

**Figure 1:**
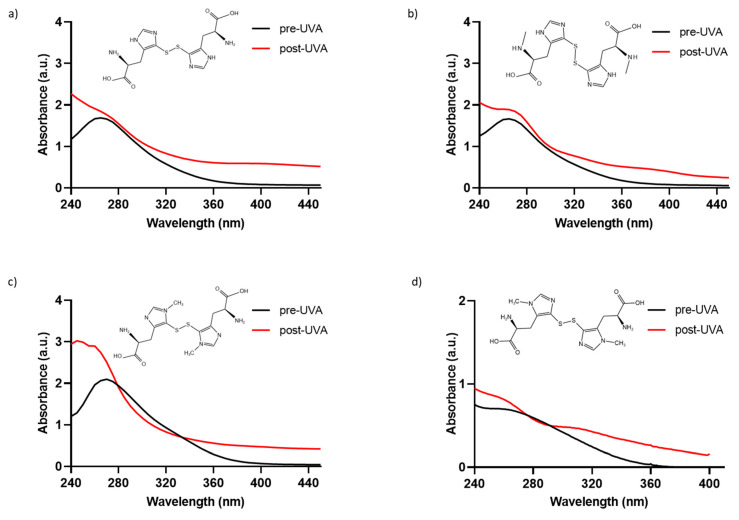
Absorbance spectra of phosphate-buffer solutions (0.5 mM) of the disulfide forms of (**a**) 5-thio, (**b**) me-5-thio, (**c**) iso-ovoA, and (**d**) 0.1 mM ovothiol A, (before (black line) and after (red line) exposure to UVA for 20 min (=~540 kJ/m^2^). The chemical structures of the disulfide forms are shown in each panel.

To determine if the chemically synthesized compounds exhibit the characteristic absorption profile of the natural compound ovothiol A, solutions of 0.5 mM of 5-thio, me-5-thio, and iso-ovoA, were analyzed to characterize the absorbance spectrum in the 240–440 nm range (Figure 1a–c). For comparison, the natural compound is reported in Figure 1d, which exhibits maximum absorption at around 254–260 nm in its disulfide form. The spectral profiles of the three synthetic compounds in their disulfide form are all similar, with their maximum absorption peaks being between 264 and 266 nm (Figure 1a–c), and they are comparable to that of the natural ovothiol A (Figure 1d).

To assess their photostability, the three compounds were exposed to UVA irradiation for 20 min, and their absorbance spectra were monitored. Only the spectra at the exposure time of UVA for 20 min, equivalent to ~540 kJ/m^2^, are shown, since longer exposure times did not induce further changes in the absorbance spectra. As shown in Figure 1a–c, UVA exposure induces a change in the absorption spectrum of all three synthetic molecules since the evident peak at around 265 nm disappears, followed by a concomitant increase in absorbance from 260 nm onwards due to the homolytic cleavage of the disulfide bond [33]. In detail, at 0.5 mM, the absorbance in the UVA range (320–400 nm) increases, reaching approximately 1 AU. Also, for the natural compound, we observed an increase in absorbance in the UVA range after UVA exposure (Figure 1d). The increase in the UVA range is slightly lower for me-5-thio. The calculated Bond Dissociation Energy (BDE) for me-5-thio is 118.36 kJ/mol compared to 5-thio (111.94 kJ/mol) and iso-ovoA (99.44 kJ/mol), more similar to the value of the natural ovothiol A (115.24 kJ/mol) [33]. The calculated S-S bond length of me-5-thio (2.14 Å) is slightly shorter than that of 5-thio (2.20 Å) and iso-ovoA (2.19 Å) and closer to that of the natural ovothiol A (2.15 Å) [33]. For all subsequent experiments, the disulfide forms were always pre-irradiated for 20 min to obtain the reduced forms with a higher absorbance capacity.

### 2.2. Shielding Effect of Marine Ovothiol Chemical Analogs on HDF Viability upon UVA Exposure

**Figure 2:**
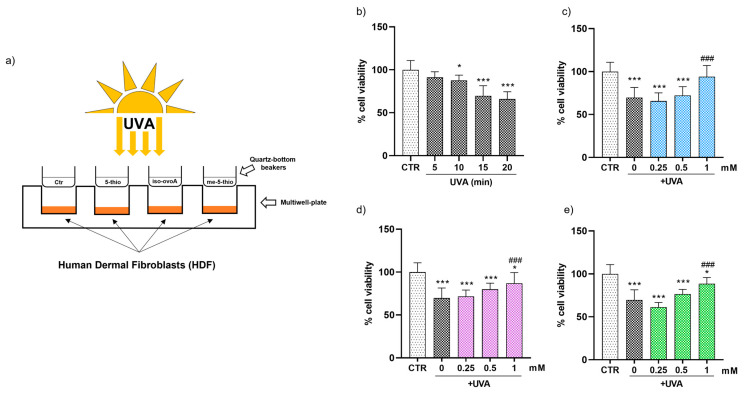
(**a**) Representative scheme of the experimental set-up for UVA exposure using marine ovothiol analogs as shielding agents. (**b**) Time course of cell viability of HDFs exposed to UVA, assessed using the Presto Blue Assay. The effect of increasing concentrations (0.25–1 mM) of, (**c**) 5-thio (blue bars), (**d**) 5-me-thio (purple bars), and (**e**) iso-ovoA (green bars) used as shielding agents on cell viability determined after exposure to UVA for 15 min. Statistical significance was calculated with an ordinary one-way ANOVA using Tukey’s multiple comparison test. * *p* < 0.05, *** *p* < 0.0001 vs. CTR; ### *p* < 0.0001 vs. 0, i.e., in the absence of compounds exposed to UVA. CTR = non-irradiated cells used as the negative control.

To explore the shielding effects of 5-thio, me-5-thio, and iso-ovoA on HDFs, we used the experimental set-up shown in Figure 2a, adding solutions of these compounds into custom-made quartz-bottom beakers placed on top of the cell culture wells containing HDFs, therefore not directly making contact with the cells. At first, HDFs were subjected to different UVA exposure times (5, 10, 15 and 20 min) to establish the ideal UVA stress condition. For this purpose, the PrestoBlue reagent, a cell-permeable resazurin-based solution, was used as a cell viability indicator of the reducing power of living cells to measure cell proliferation quantitatively. The results reported in Figure 2b show that, from 10 min onwards, there was a gradual and significant decrease in cell viability after UVA exposure: hence for subsequent experiments, 15 min was selected as a time point that induces a moderate but significant reduction (~20%) in cell viability without excessively damaging the cells. Subsequently, three different concentrations (0.25, 0.5 and 1 mM) of pre-irradiated 5-thio, me-5-thio, and iso-ovoA were used to determine their possible shielding effect on HDFs exposed to UVA. Figure 2c–e clearly shows that, in the presence of all three compounds, there is a steady increase in cell viability which becomes significant at 1 mM concentration compared to the control cells exposed to UVA for 15 min in their absence. This result indicates that the compounds can shield against UVA rays, preventing the cells from impaired cell viability.

**Figure 3:**
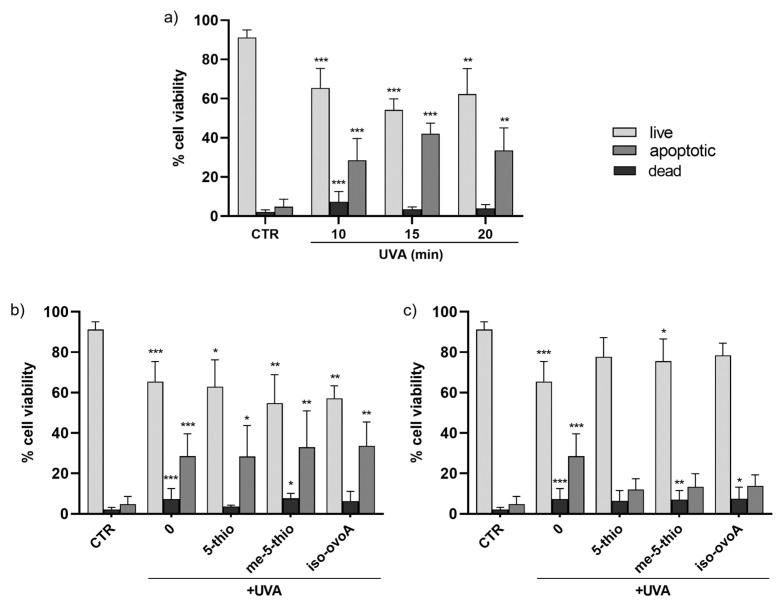
(**a**) Time course of cell viability in HDFs, measured by flow cytometry using the ViaCount probe, after exposure to UVA for 10, 15, and 20 min. (**b**) Effects on HDFs cell viability in the presence of 0.5 mM 5-thio, 5-me-thio, and iso-ovoA used as shielding agents and measured after UVA exposure for 10 min. (**c**) Effects of the presence of the tested compounds at 1 mM on HDFs viability. Statistical significance was calculated with Kruskal–Wallis test using Dunn’s multiple comparison test. * *p* < 0.05, ** *p* < 0.01, *** *p* < 0.0001 vs. CTR. CTR = non-irradiated cells used as negative control.

These results were further confirmed using a flow cytometric approach with the aid of the ViaCount reagent containing two DNA-binding dyes which, based on their differential permeabilities, are able to distinguish viable, apoptotic, and non-viable cells. This methodology gives a more precise evaluation on the viability of the cell population in a single measurement compared to assays that use standard plate readers that measure metabolic markers to estimate the number of viable cells in culture, such as the Presto Blue assay, which are, however, useful for a first rapid and inexpensive screening of cell viability. Figure 3a shows that the number of viable cells upon UVA exposure for 10, 15, and 20 min is reduced by ~20% while the number of apoptotic cells increases by ~20–30% compared to non-exposed cells. The number of dead cells also significantly increases after 10 min of UVA irradiation; however, at longer exposure times the number of dead cells is apparently reduced, likely due to the fact that, when cells are dead, they are no longer easily detectable as they disintegrate into debris. The addition of 0.5 mM ovothiol analogs does not significantly alter the effect of UVA exposure on HDF viability and apoptotic cells (Figure 3b). However, the addition of 1 mM 5-thio, me-5-thio, and iso-ovoA (Figure 3c), while not exhibiting a significant increase in the number of live cells, slightly reduced the percentage of apoptotic cells following UVA exposure compared to irradiated cells without compounds. On the other hand, the levels of dead cells in the presence of the compounds did not significantly differ from those of UVA-exposed cells, except in the presence of 5-thio, where the results were more similar to that of the unexposed cells.

### 2.3. Protective Effect of Marine Ovothiol Analogs Against UVA-Induced ROS in HDF

**Figure 4:**
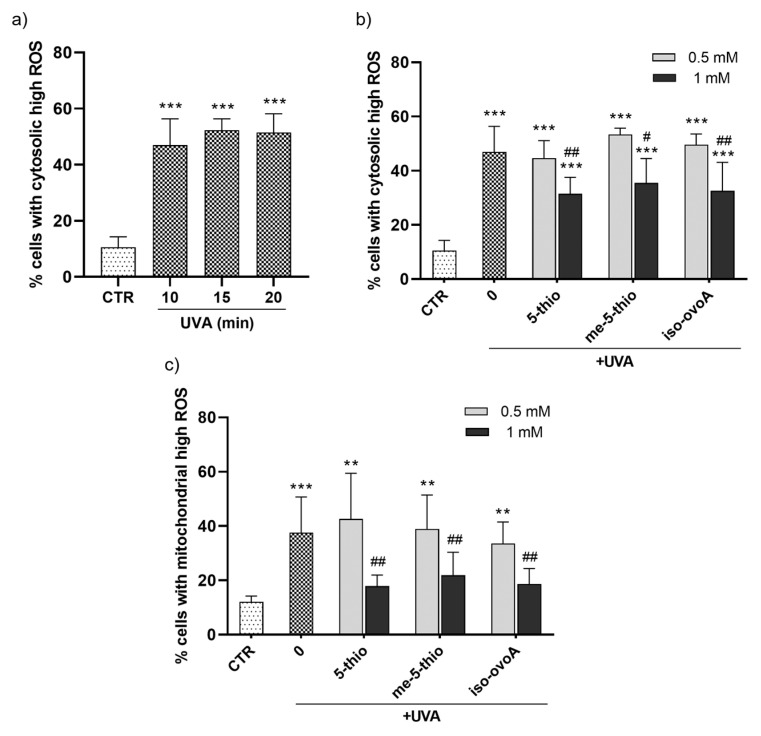
(**a**) Time course of the production of cytosolic high ROS in HDFs measured after UVA exposure for 10, 15, and 20 min using a flow cytometric assay with the ROS-sensitive probe CM-H_2_DCFDA. (**b**) Percentage of cells with cytosolic high ROS measured after UVA exposure for 10 min in the presence of 0.5 mM and 1 mM 5-thio, me-5-thio, iso-ovoA used as shielding agents. (**c**) Percentage of cells with mitochondrial high ROS assessed in the same conditions using the probe MitoSOX Red. The statistical significance was calculated with an ordinary one-way ANOVA using Tukey’s multiple comparison test. ** *p* < 0.01, *** *p* < 0.0001 vs. CTR; # *p* < 0.05, ## *p* < 0.01 vs. 0, i.e., in the absence of compounds exposed to UVA. CTR = non-irradiated cells used as negative control.

To evaluate the potential protective effect of marine ovothiol analogs against the production of cytosolic ROS after UVA exposure, a flow cytometric assay using the ROS-sensitive probe, dichlorofluorescein diacetate (CM-H_2_DCFDA), was performed. Initially, a time course experiment was set to determine the required UVA exposure time necessary to achieve significant cytosolic ROS production. The ROS levels significantly increased after UVA exposure for 10 min (Figure 4a). Subsequently, the shielding effect of 0.5 mM and 1 mM 5-thio, me-5-thio, and iso-ovoA against UVA-induced ROS production in HDFs was investigated according to the set-up shown in Figure 2a. Remarkably, all three compounds significantly shielded UVA rays at a 1 mM concentration, leading to a decrease in cytosolic ROS levels (Figure 4b), albeit not to the level of the non-irradiated cells (negative control). At 0.5 mM, the compounds did not significantly counteract ROS production in HDFs. Since mitochondria are the most important subcellular sites of ROS production in mammalian cells, the potential of 5-thio, me-5-thio, and iso-ovoA to protect against mitochondrial ROS production upon UVA exposure for 10 min was investigated by a flow cytometric assay using the probe MitoSOX Red. In the presence of all three compounds, at 1 mM concentration, a significant decrease (>50%) in mitochondrial ROS levels compared to cells exposed to UVA in their absence was observed, indicating a relevant shielding effect (Figure 4c). At 0.5 mM, the compounds did not lead to significant mitochondrial ROS reduction in HDFs.

## 3. Discussion

Artificial (man-made) UV filters are currently used in sunscreen products to protect the skin from sunburns, photoaging, and photocarcinogenesis [6,7,40]. However, the efficacy and safety of some of them are hampered by their photoinstability, toxicity, and damage to marine ecosystems, especially coral reefs [5,10,11,12,13,14]. Terrestrial and marine organisms have evolved protective mechanisms against the deleterious side effects of oxidative stress and UV. Hence, naturally occurring compounds with UV-screening, antioxidant, and anti-inflammatory properties are drawing considerable attention due to their peculiar structural properties, especially the conjugated π system, whose electrons can absorb photons’ energy and translocate from a ground state to an excited one [41]. Compared to artificial ones, some natural UV filters with UV-absorptive capacities are limited by low specific extinction values and by the inability to be incorporated into large-scale sunscreen cosmetic applications [16]. However, several studies have documented that natural components exert their photoprotective effects (for example, improving skin elasticity and hydration and preventing wrinkle formations) through their antioxidant effects and by regulation of UV-induced skin inflammation and aging [42,43,44,45].

In this work, we analyzed the UV-shielding effect of three synthetic L-5-sulfanyl histidine disulfide derivatives, 5-thio, iso-ovo A, and me-5-thio, inspired by their marine natural counterparts, ovothiols, whose purification from the most abundant natural sources, e.g., sea urchin eggs, mussels, and microalgae, represents a difficult task [27,46]. Therefore, the screening of the chemical and biological properties of synthetic derivatives and the comparison with natural counterparts represent an interesting challenge for future cosmeceutical applications. Ovothiols and their analogs are endowed with a unique chemical structure, characterized by an imidazole ring with aromatic properties and a peculiar thiol group which can be oxidized to its disulfide form (see Figure 1). This typical chemical structure confers on these molecules the ability to absorb UV light mainly in the range of 254–320 nm, protecting against UVC and UVB rays (280–320 nm). Our results, consistent with previous studies [33], confirmed that the UVA shielding properties of 5-thio, iso-ovo A, and me-5-thio in their disulfide form increase after exposure to UVA. This behavior may be ascribed to the partial cleavage of the disulfide bond, leading to the corresponding reduced forms of the compounds without affecting the stability of the molecules. Indeed, we have previously shown that 5-thio and iso-ovoA, provided in their oxidized forms, undergo a reduction in the disulfide bond upon UVA exposure, leading to a higher absorption profile in the UVA range (320–400 nm) [33], an interesting property of potential UV filters to be used in sunscreens. Our new data indicate that, for me-5-thio, the disulfide bond is more stable than the disulfide of 5-thio and iso-ovoA and therefore more resistant to homolytic cleavage induced by UVA. Interestingly, the strength of the disulfide bond of me-5-thio is very similar to that of the natural compound ovothiol A. The differences in the stability of the disulfide forms are likely due to the stabilization effect of the methyl group on the lateral chain of me-5-thio. Our data confirm that, besides 5-thio and iso-ovoA, me-5-thio in its reduced form can also effectively shield UVA rays compared to the oxidized form, reaching a greater absorption value, which is likely responsible for preventing the in vitro UVA-induced oxidative damage. The reasons for the difference in absorption profile between the reduced and oxidized forms remain obscure. However, we can hypothesize that the intermolecular interactions among the sulfur-containing histidine compounds change in the two states, and this could be responsible for the different absorption profiles. For example, more structural constraints can be envisaged for the oxidized forms; on the other hand, the reduced forms can undergo stabilization effects due to electronic resonance. Overall, these features indicate that L-5-sulfanyl histidine derivatives display UVA-protective properties, similarly to MAAs, which are currently considered the strongest UVA-absorbing amino acids in nature due to their ability to absorb light both in the UVA (315–400 nm) and UVB (280–315 nm) range [17,18,47]. However, compared to MAAs, the molar extinction coefficients of L-5-sulfanyl histidine derivatives are lower [47].

More interestingly, shielding experiments performed on HDFs show that 5-thio, me-5-thio, and iso-ovoA following pre-irradiation with UVA are efficient UV-filters for skin cells at 1 mM. At this concentration, the three compounds filter out UVA rays and prevent the production of both cytosolic and mitochondrial ROS in HDFs, confirming the potential role of these compounds in preventing UVA-induced oxidative damage. In particular, oxidative damage caused by mitochondrial ROS production is considered the molecular basis of multiple pathophysiological conditions, including skin inflammation and aging [48]. As the largest organ in the body, and as the interface with the outside world, the skin relies on a continuous supply of cells to be an effective, protective barrier. The cells present in all three skin layers rely heavily on mitochondrial function for various essential processes, including growth, repair, and structural integrity, powering cellular turnover, thereby influencing the overall skin structure and function. Not surprisingly, mitochondria are the primary organelles affected during chronological and UV-induced skin aging, where the phenotypic manifestations, such as wrinkles, loss of elasticity, age spots, as well as increased susceptibility to chronic skin conditions and even skin cancer, are the direct consequence of mitochondrial dysfunction [49]. Also, deletions and other aberrations in mitochondrial DNA (mtDNA) are frequent in photoaged skin and skin cancer lesions. Some common and rare skin disorders have mitochondrial involvement and include dermal manifestations of primary mitochondrial diseases as well as congenital skin diseases caused by damaged mitochondria [48]. Therefore, managing oxidative stress through antioxidants or lifestyle changes, such as reducing UV exposure by the use of sunscreens, may help mitigate these effects and improve long-term skin health. In this context, the protective effect of the three ovothiol analogs against the reduction in cell viability and the induction of apoptotic processes induced by UVA exposure in HDFs suggests their potential use for developing novel cosmetic formulations, which, combined with other natural UV filters, could have a positive impact on the environment and human health. Their safety for the environment can be envisaged from the findings that several marine organisms, such as sea urchins, corals, and microalgae, produce ovothiols as protective molecules against different environmental stressors, like UVA [34,35]. However, our previous study confirms no toxicity for 5-thio towards human skin cells [32]. Curiously, ovothiols have been recently found in the lenses of fish, again suggesting specialized UVA protective properties in the eyes [36] and potential applications to delay or prevent the aging and the formation of cataracts in human lenses [37].

## 4. Materials and Methods

### 4.1. Reagents

Cell culture reagents were obtained from Euroclone (Euroclone S. p. A., Pero, MI, Italy) and Merck (Merck KGaA, Darmstadt, Germany). PrestoBlue (Cat. no. A13262), MitoSOX Red (Cat. no. M36008), and CM-H_2_DCFDA (Cat. no. C6827) were purchased from Thermo Fisher Scientific (Thermo Fisher Scientific, Waltham, MA, USA), whereas Guava ViaCount Reagent was purchased from Cytek Biosciences (Fremont, CA, USA). Iso-ovoA, 5-thio and me-5-thio were kindly donated by Jean Claude Yadan, Tetrahedron Company (Paris, France), and prepared as described in [38], while ovothiol A was purified from *Paracentrotus lividus* eggs, as described in Russo et al. 2014 [39].

### 4.2. UV-Vis Spectrophotometric Measurements

Stock solutions (10 mM) of the synthetic and natural compounds were prepared in deionized water, as reported in [33]. In short, appropriate amounts of phosphate-buffered saline (PBS) were then added to reach the final concentrations of 0.5 mM for 5-thio, me-5-thio, and iso-ovoA. The solutions (200 μL) were then transferred to a UV-transparent 96-well plate and an absorption spectrum of between 240 and 450 nm was determined on a multi-plate reader (BIO-TEK Synergy HT, BioTEK Instruments Inc., Winooski, VT, USA) before and after UVA exposure for 20 min. The UVA source is the same as previously described in [33].

### 4.3. Computational Methods

The homolytic BDEs of the me-5-thio were calculated according to our previously tested set-up protocol [33]. Gaussian 16 [50] was used for all DFT calculations. The equilibrium geometries of me-5-thio (RS-SR) and the free radicals produced (RS^•^) were fully optimized by employing the hybrid density functional B3LYP together with a 6–31 G (d,p) basis set, whose accuracy for optimization and frequency calculations of this class of organic compounds has already been assessed [33,51,52,53]. All optimizations were checked for convergence with an energy minimum, which included checking for convergence and ensuring that the resulting structure had no imaginary vibrational frequencies and was in the lowest minimum energy state. Standard thermodynamic parameters at 298 K, including zero-point energy (ZPE) corrections, were obtained by vibrational frequency calculations. The varied conformers were fully optimized to find the energy minima. The BDE is the total energy required for the homolytic cleavage of a specific bond, in this case, the disulfide bond (S-S) of the oxidized thiol (RS-SR) compounds. The BDE is thus the enthalpy change in the reaction RS-SR → 2RS^•^, and the values reported are calculated following the widely used protocol of Wright, as explained in our previous work [33].

### 4.4. Cell Culture

Primary human dermal fibroblast (HDFs) cells [54] were purchased from the Istituto Zooprofilattico Sperimentale (Brescia, Italy) and derived from pooled samples of female donors (40 years). HDFs were grown at 37 °C in CO_2_ under a humidified atmosphere in a Thermo Scientific Heracell 150i CO_2_ Incubator (Thermo Fisher Scientific, Waltham, MA, USA). A Minimum Essential Medium (MEM) supplemented with 10% (*v*/*v*) fetal bovine serum (FBS), 2 mM stable glutamine, 1% penicillin (10,000 U/mL), 1% streptomycin (10 mg/mL), and 1% amphotericin (250 µg/mL) was used for the cell culture. The complete medium was replaced every two days. For the experiments, cells were seeded in 96-well plates or 24-well plates at an optimal density of 3 × 10^4^ and 15 × 10^4^ cells/cm^2^, respectively.

### 4.5. UVA Exposure Treatment

For UVA exposure, the HDFs were first washed, covered with a thin layer of PBS, and irradiated using the same experimental set-up described in [33]. To determine the UV-shielding capacity, the solutions (500 µL) of pre-irradiated thiol compounds (exposed to 20 min of UVA) were transferred into custom-made quartz-bottom beakers and placed on top of each cell culture well (see Figure 2a). The UVA dose was measured with a UV Power Pack Radiometer (EIT Inc., Sterling, VA, USA), and consistency in the UV dose received by the samples at the same distance from the light source was ensured before each exposure by measuring the intensity of the UV light emitted with the radiometer. As control, an empty beaker containing only PBS was used, i.e., without any UV-absorbing compounds to observe the effects of UVA irradiation on the underlying HDFs. For the negative control, HDFs were not exposed to UVA, i.e., the corresponding cell culture wells were covered with an opaque material (specifically, a sheet of dark cardboard) to effectively obstruct UV radiation, thus preventing direct exposure to harmful UV wavelengths and minimizing any potential photodamage to the cells.

### 4.6. Cell Viability Assay

The cell viability assay was assessed using a Presto Blue Cell Viability Reagent. After reaching the appropriate confluence, the 96-well plate covered on top with a 2 mm-thick quartz slab of the same dimensions as the cell culture plate was exposed to UVA for 5, 10, 15, and 20 min (respectively corresponding to 135, 270, 405 and 540 kJ/m^2^). Once the desired irradiation time was chosen, the experiments were performed using 0.25 mM, 0.5 mM, and 1 mM pre-irradiated solutions of each marine-inspired thiol compound.

### 4.7. Cell Viability and Cytosolic ROS Assay

Guava ViaCount Reagent (Cytek Biosciences) and CM-H_2_DCFDA, a chloromethyl derivative of H_2_DCFDA, were simultaneously used to measure, respectively, the cell viability and total cytosolic ROS levels as described in [55,56] with some modifications. A preliminary experiment was performed at different UVA exposure times for choosing a sub-lethal dose of irradiation. Solutions at 0.5 mM and 1 mM of the pre-irradiated thiol compounds were then used as shielding agents. Briefly, after UVA exposure, a 1 µM working solution of CM-H_2_DCFDA in PBS was added to each 24-well plate and cells were incubated in the dark for 15 min at 37 °C. After trypsinisation, cells were harvested and then centrifuged at 500× *g* for 5 min. The cell pellet was resuspended in approximately 150 µL of a ViaCount/PBS solution (1:5). The analyses were performed on a Guava Easycite flow cytometer (Cytek Biosciences, Cytek Biosciences B.V., Fremont, CA, USA) using an excitation wavelength of 488 nm. Emissions were recorded using the green channel for carboxy-DCF and the red and yellow channels for the ViaCount dye. The analyses were recorded on an average of 5000 cells from each sample, and the results are expressed as the percentage of cells with high cytosolic ROS. Experiments were carried out at least in triplicate, and the results were analyzed using guavaSoft^TM^ software version 3.1.1.

### 4.8. Mitochondrial Superoxide Anion Assay

Mitochondrial superoxide production was evaluated using a MitoSOX Red fluorescent probe [57]. After reaching the appropriate confluence, cells were exposed to UVA as previously described above, using the reduced forms of thiol compounds at 0.5 mM and 1 mM as shielding agents. A 2.5 µM working solution of MitoSOX Red was prepared in PBS and then added to each 24-well plate and incubated in the dark for 15 min at 37 °C. After harvesting as described above, the cells were resuspended in approximately 150 µL PBS. The analyses were conducted on the Guava EasyCyte flow cytometer using an excitation wavelength of 488 nm. Emissions were recorded using the yellow channel for MitoSOX Red. The fluorescence intensity was recorded on an average of 5000 cells from each sample, and the results are expressed as the percentage of cells with high mitochondrial ROS.

### 4.9. Statistical Analysis

All experiments were performed at least three times in duplicate and conducted in different experimental sessions. Data are expressed as the mean ± standard deviation. Statistical analyses were performed using a GraphPad Prism 8.4.2 Kruskal–Wallis test and ordinary one-way analysis of variance (ANOVA) software. All the data were subjected to normality tests using Shapiro–Wilk and D’Agostino and Pearson analyses, while the effect size was calculated using the Eta Squared test. These are all reported in the Supplementary File (S1). Values of *p* < 0.05 and *p* < 0.01 were considered statistically significant (Dunn’s and Tukey’s multiple-comparison tests).

## 5. Conclusions

The currently available commercial UV filters raise several health and environmental concerns. The study of photoprotective properties of natural products and their synergistic effects could partially reduce the overall reliance on synthetic physical or chemical UV filters. This study provides the foundations to further explore the UVA protective effects of ovothiols analogs which, in the future, may be combined with other marine-derived natural products, such as MAAs, to enhance the sun protection factor and create eco-safe, broad-spectrum sunscreens. However, although these compounds show good potential, their integration in sunscreen formulations is not straightforward, and further research and development are required to overcome issues related to their scalability, technological viability and competitiveness, and formulation issues such as compatibility and stability, as is the case with all nature-derived compounds [58].

Future studies will be devoted to assessing the effects of such compounds in direct contact with HDFs under UVA exposure to confirm their safety for human skin applications. Previous toxicity tests on the natural ovothiol A and 5-thio proved that these compounds were not cytotoxic for keratinocytes, at least up to 0.2 mM, but instead induced an anti-inflammatory response [32]. Generally, the compounds are administered to cell cultures in their oxidized forms; however, they are known to be reduced by the intracellular GSH, thus acting as antioxidants [27]. Interestingly, a natural product similar to ovothiols, ergothioneine, has been reported to exert dermo-protective activity through the induction of Nrf2/ARE-mediated antioxidant genes in UVA-irradiated human keratinocytes [59].

## Data Availability

Data are contained within the article.

## Supplementary Materials

The following supporting information can be downloaded at: https://www.mdpi.com/article/10.3390/md23020057/s1. Supplementary file S1: The *p* values and effect size related to the statistical analysis of this work.
