# Supplementary material for: Characterizing the Ultraviolet (UV) Screening Ability of L-5-Sulfanylhistidine Derivatives on Human Dermal Fibroblasts
Source: Mar Drugs. 2025 Jan 24;23(2):57. doi: 10.3390/md23020057 (PMC11857345; doi:10.3390/md23020057)
Supplement: Supplementary file 1 [file marinedrugs-23-00057-s001.zip › marinedrugs-3390164-supplementary.pdf]

Supplementary File S1

p-values and effect size reported for the data of Figures 2, 3 and 4

FIGURE 2

Figure 2B	p-value
CTR vs. 10	0.0281
CTR vs. 15	<0.0001
CTR vs. 20	<0.0001

Figure 2C	p-value
CTR vs. 0	<0.0001
CTR vs. 0.25	<0.0001
CTR vs. 0.5	<0.0001
0 vs. 1	<0.0001

Figure 2D	p-value
CTR vs. 0	<0.0001
CTR vs. 0.25	<0.0001
CTR vs. 0.5	<0.0001
CTR vs. 1	0.0315
0 vs. 1	0.0024

Figure 2E	p-value
CTR vs. 0	<0.0001
CTR vs. 0.25	<0.0001
CTR vs. 0.5	<0.0001
CTR vs. 1	0.033
0 vs. 1	<0.0001

Effect size (η^2)	
Fig. 2B	0.66
Fig. 2C	0.65
Fig. 2D	0.63
Fig. 2E	0.68

FIGURE 3

Figure 3A	
**Live Cells**	**p-value**
CTR vs. 10	<0.0001
CTR vs. 15	<0.0001
CTR vs. 20	0.0014
**Dead Cells**	**p-value**
CTR vs. 10	<0.0001
**Apoptotic Cells**	**p-value**
CTR vs. 10	<0.0001
CTR vs. 15	<0.0001
CTR vs. 20	0.0007

Figure 3B	
**Live Cells**	**p-value**
CTR vs. 0	<0.0001
CTR vs. 5-thio	0.0117
CTR vs. me-5-thio	0.0007
CTR vs. iso-ovoA	0.003
**Dead Cells**	**p-value**
CTR vs. 0	<0.0001
CTR vs. me-5-thio	0.0202
**Apoptotic Cells**	**p-value**
CTR vs. 0	<0.0001
CTR vs. 5-thio	0.047
CTR vs. me-5-thio	0.0084
CTR vs. iso-ovoA	0.0067

Figure 3C	
**Live Cells**	**p-value**
CTR vs. 0	<0.0001
CTR vs. me-5-thio	0.0401
**Dead Cells**	**p-value**
CTR vs. 0	<0.0001
CTR vs. me-5-thio	0.0088
CTR vs. iso-ovoA	0.0158
**Apoptotic Cells**	**p-value**
CTR vs. 0	<0.0001

Effect size (η^2)		
Fig. 3A	Live Cells	0.71
	Dead Cells	0.43
	Apoptotic Cells	0.72
Fig. 3B	Live Cells	0.70
	Dead Cells	0.50
	Apoptotic Cells	0.63
Fig. 3C	Live Cells	0.66
	Dead Cells	0.43
	Apoptotic Cells	0.72

FIGURE 4

Figure 4A	p-value
CTR vs. 10	<0.0001
CTR vs. 15	<0.0001
CTR vs. 20	<0.0001

Figure 4B	
**0.5 mM**	**p-value**
CTR vs. 0	<0.0001
CTR vs. 5-thio	<0.0001
CTR vs. me-5-thio	<0.0001
CTR vs. iso-ovoA	<0.0001
**1 mM**	**p-value**
CTR vs. 0	<0.0001
CTR vs. 5-thio	<0.0001
CTR vs. me-5-thio	<0.0001
CTR vs. iso-ovoA	<0.0001
0 vs. 5-thio	0.0008
0 vs. me-5-thio	0.0213
0 vs. iso-ovoA	0.002

Figure 4C	
**0.5 mM**	**p-value**
CTR vs. 0	<0.0001
CTR vs. 5-thio	0.0002
CTR vs. me-5-thio	0.0009
CTR vs. iso-ovoA	0.0091
**1 mM**	**p-value**
CTR vs. 0	<0.0001
0 vs. 5-thio	0.0002
0 vs. me-5-thio	0.0029
0 vs. iso-ovoA	0.0003

Effect size (η^2)		
**Fig. 4A**		0.89
**Fig. 4B**	**0.5 mM**	0.88
	**1 mM**	0.80
**Fig. 4C**	**0.5 mM**	0.71
	**1 mM**	0.65
